# The Rhodoexplorer Platform for Red Algal Genomics and Whole-Genome Assemblies for Several *Gracilaria* Species

**DOI:** 10.1093/gbe/evad124

**Published:** 2023-07-22

**Authors:** Agnieszka P Lipinska, Stacy A Krueger-Hadfield, Olivier Godfroy, Simon M Dittami, Lígia Ayres-Ostrock, Guido Bonthond, Loraine Brillet-Guéguen, Susana Coelho, Erwan Corre, Guillaume Cossard, Christophe Destombe, Paul Epperlein, Sylvain Faugeron, Elizabeth Ficko-Blean, Jessica Beltrán, Emma Lavaut, Arthur Le Bars, Fabiana Marchi, Stéphane Mauger, Gurvan Michel, Philippe Potin, Delphine Scornet, Erik E Sotka, Florian Weinberger, Mariana Cabral de Oliveira, Marie-Laure Guillemin, Estela M Plastino, Myriam Valero

**Affiliations:** Department of Algal Development and Evolution, Max Planck Institute for Biology Tubingen, Tubingen, Germany; CNRS, UMR 8227, Laboratory of Integrative Biology of Marine Models, Sorbonne Université, Station Biologique de Roscoff, Roscoff, France; Department of Biology, University of Alabama at Birmingham, Alabama, USA; CNRS, UMR 8227, Laboratory of Integrative Biology of Marine Models, Sorbonne Université, Station Biologique de Roscoff, Roscoff, France; CNRS, UMR 8227, Laboratory of Integrative Biology of Marine Models, Sorbonne Université, Station Biologique de Roscoff, Roscoff, France; Departamento de Botânica, Instituto de Biociências, Universidade de São Paulo, SP, Brazil; Hortimare, Breeding and Propagating Seaweed, Heerhugowaard, The Netherlands; Institute for Chemistry and Biology of the Marine Environment (ICBM), Carl von Ossietzky University Oldenburg, Wilhelmshaven, Germany; CNRS, UMR 8227, Laboratory of Integrative Biology of Marine Models, Sorbonne Université, Station Biologique de Roscoff, Roscoff, France; CNRS, Sorbonne Université, FR2424, ABiMS-IFB, Station Biologique, Roscoff, France; Department of Algal Development and Evolution, Max Planck Institute for Biology Tubingen, Tubingen, Germany; CNRS, Sorbonne Université, FR2424, ABiMS-IFB, Station Biologique, Roscoff, France; Department of Algal Development and Evolution, Max Planck Institute for Biology Tubingen, Tubingen, Germany; CNRS, Sorbonne Université, Pontificia Universidad Católica de Chile, Universidad Austral de Chile, IRL 3614, Evolutionary Biology and Ecology of Algae, Station Biologique de Roscoff, Roscoff, France; Department of Algal Development and Evolution, Max Planck Institute for Biology Tubingen, Tubingen, Germany; CNRS, Sorbonne Université, Pontificia Universidad Católica de Chile, Universidad Austral de Chile, IRL 3614, Evolutionary Biology and Ecology of Algae, Station Biologique de Roscoff, Roscoff, France; Núcleo Milenio MASH, Facultad de Ciencias Biológicas, Pontificia Universidad Católica de Chile, Santiago, Chile; CNRS, UMR 8227, Laboratory of Integrative Biology of Marine Models, Sorbonne Université, Station Biologique de Roscoff, Roscoff, France; CNRS, Sorbonne Université, Pontificia Universidad Católica de Chile, Universidad Austral de Chile, IRL 3614, Evolutionary Biology and Ecology of Algae, Station Biologique de Roscoff, Roscoff, France; Núcleo Milenio MASH, Facultad de Ciencias Biológicas, Pontificia Universidad Católica de Chile, Santiago, Chile; CNRS, Sorbonne Université, Pontificia Universidad Católica de Chile, Universidad Austral de Chile, IRL 3614, Evolutionary Biology and Ecology of Algae, Station Biologique de Roscoff, Roscoff, France; CNRS, Sorbonne Université, FR2424, ABiMS-IFB, Station Biologique, Roscoff, France; CNRS, Institut Français de Bioinformatique, IFB-core, Évry, France; Departamento de Botânica, Instituto de Biociências, Universidade de São Paulo, SP, Brazil; CNRS, Sorbonne Université, Pontificia Universidad Católica de Chile, Universidad Austral de Chile, IRL 3614, Evolutionary Biology and Ecology of Algae, Station Biologique de Roscoff, Roscoff, France; CNRS, UMR 8227, Laboratory of Integrative Biology of Marine Models, Sorbonne Université, Station Biologique de Roscoff, Roscoff, France; CNRS, UMR 8227, Laboratory of Integrative Biology of Marine Models, Sorbonne Université, Station Biologique de Roscoff, Roscoff, France; CNRS, UMR 8227, Laboratory of Integrative Biology of Marine Models, Sorbonne Université, Station Biologique de Roscoff, Roscoff, France; Department of Biology, College of Charleston, Charleston, South Carolina, USA; Marine Ecology Division, GEOMAR Helmholtz-Zentrum für Ozeanforschung, Kiel, Germany; Departamento de Botânica, Instituto de Biociências, Universidade de São Paulo, SP, Brazil; CNRS, Sorbonne Université, Pontificia Universidad Católica de Chile, Universidad Austral de Chile, IRL 3614, Evolutionary Biology and Ecology of Algae, Station Biologique de Roscoff, Roscoff, France; Núcleo Milenio MASH, Facultad de Ciencias, Instituto de Ciencias Ambientales y Evolutivas, Universidad Austral de Chile, Valdivia, Chile; Centro FONDAP de Investigación de Ecosistemas Marinos de Altas Latitudes (IDEAL), Valdivia, Chile; Departamento de Botânica, Instituto de Biociências, Universidade de São Paulo, SP, Brazil; CNRS, Sorbonne Université, Pontificia Universidad Católica de Chile, Universidad Austral de Chile, IRL 3614, Evolutionary Biology and Ecology of Algae, Station Biologique de Roscoff, Roscoff, France

**Keywords:** evolution, ecology, omics, ploidy, Rhodophyta

## Abstract

Macroalgal (seaweed) genomic resources are generally lacking as compared with other eukaryotic taxa, and this is particularly true in the red algae (Rhodophyta). Understanding red algal genomes is critical to understanding eukaryotic evolution given that red algal genes are spread across eukaryotic lineages from secondary endosymbiosis and red algae diverged early in the Archaeplastids. The Gracilariales is a highly diverse and widely distributed order including species that can serve as ecosystem engineers in intertidal habitats and several notorious introduced species. The genus *Gracilaria* is cultivated worldwide, in part for its production of agar and other bioactive compounds with downstream pharmaceutical and industrial applications. This genus is also emerging as a model for algal evolutionary ecology. Here, we report new whole-genome assemblies for two species (*Gracilaria chilensis* and *Gracilaria gracilis*), a draft genome assembly of *Gracilaria caudata*, and genome annotation of the previously published *Gracilaria vermiculophylla* genome. To facilitate accessibility and comparative analysis, we integrated these data in a newly created web-based portal dedicated to red algal genomics (https://rhodoexplorer.sb-roscoff.fr). These genomes will provide a resource for understanding algal biology and, more broadly, eukaryotic evolution.

**Significance**

The Gracilariales are an ecologically and economically important red algal order found throughout the coastal regions of the world. Understanding the biology, ecology, and evolution of species in this order, and that of red algae more broadly, has been hampered by the limited phylogenetic coverage of genomic resources. Here, we present whole-genome assemblies and gene annotations for four *Gracilaria* species that will serve as a key resource for algal research on evolution, ecology, biotechnology, and aquaculture.

## Introduction

Red algae (Rhodophyta) represent a lineage of photosynthetic eukaryotes in the Archaeplastids that diverged from green algae around 1,700 Ma (Yang et al. 2016). Within the Rhodophyta, the Cyanidiophyceae were the earliest to diverge ∼1,200 Ma, while the Florideophyceae diverged more recently (i.e., 412 Ma; Yang et al. 2016) and constitute the most speciose group (Graham et al. 2016). In this context, the genomic resources currently available (supplementary table S1, Supplementary Material online) represent only a fraction of the diversity of red algae, limiting our capacity to reconstruct the evolutionary history of the unique features of this group.

The Florideophyceae have a life cycle in which haploid male and female gametophytes alternate with a diploid tetrasporophyte (but see supplementary fig. S1, Supplementary Material online). Many species have “isomorphic” gametophytes and tetrasporophytes, which are hard to discern without the aid of molecular tools (e.g., sex-linked markers, Martinez et al. 1999; Guillemin et al. 2012; or microsatellites, Krueger-Hadfield et al. 2016).

Here, we focus on four *Gracilaria* (There is controversy over the systematics of *Gracilaria* Greville, but for the purposes of this paper, we consider the four species as belonging to the genus Gracilaria [sensu Lyra et al. 2021; Guiry and Guiry 2022]). species spanning roughly 170 Myr of evolution (Lyra et al. 2021). These species were chosen based on their evolutionary, ecological, and/or economic importance. Species in the genus *Gracilaria* produce agars in their cell wall (Popper et al. 2011); they can be propagated vegetatively and serve as ecosystem engineers in intertidal zone (Kain and Destombe 1995). The four taxa chosen can be divided into three clades based on their molecular divergence: 1) *Gracilaria chilensis* and *Gracilaria vermiculophylla*, 2) *Gracilaria caudata*, and 3) *Gracilaria gracilis* (Lyra et al. 2021). *Gracilaria gracilis* and *G. caudata* are evolutionarily more distinct than the phylogenetic group that contains *G. chilensis* and *G. vermiculophylla*. *Gracilaria chilensis* C.J. Bird et al. is an important crop along the Chilean coastline, where it has been both harvested and subsequently planted after a crash in natural stands likely due to overharvesting (Buschmann et al. 2001). The artificial selection for tetrasporophytes has resulted in early stages of domestication (Valero et al. 2017) and loss of sexual reproduction (Guillemin et al. 2008). *Gracilaria vermiculophylla* (Ohmi) Papenfuss is a successful invader in many of the bays and estuaries of the Northern Hemisphere (Krueger-Hadfield et al. 2017). These invasions were likely facilitated by adaptive shifts in temperature and salinity tolerance (e.g., Sotka et al. 2018) and to biofoulers (e.g., Bonthond et al. 2020), as well as the ability to fragment (Krueger-Hadfield et al. 2016). *Gracilaria caudata* J. Agardh can form dense stands in the intertidal zone (Plastino and Oliveira 1997) and has been subjected to intense harvesting pressure, leading to declines in native populations (Hayashi et al. 2014; see also Ayres-Ostrock et al. 2019). Finally, *G. gracilis* (Stackhouse) Steentoft, L.M. Irvine & Farnham is a long-lived species that inhabits tide pools along European coastlines. This species serves as model species to test hypotheses related to the evolution of sex (e.g., alternation of haploid and diploid phases in life cycles, Destombe et al. 1989, 1992, 1993; Hughes and Otto 1999; mating system and sexual selection, Richerd et al. 1993; Engel et al. 1999).

The availability of genomic and genetic resources for these four *Gracilaria* species should aid in our understanding of the evolutionary ecology of red algae in their dynamic environment, during invasions of new habitats, under cultivation practices, and in response to climate change. Moreover, these new resources will add to the existing genomic data and illuminate key processes in eukaryotic evolution. The Rhodoexplorer Red Algal Genome Database currently includes the *Gracilaria* species discussed here but will include all the high-quality genomic resources available for the Rhodophyta (e.g., genomes and transcriptomes), thereby providing a unique resource for comparative analyses.

## Results and Discussion

### Genome Assembly

Genome assembly sizes were 72 and 76 Mb for *G. gracilis* and *G. chilensis*, respectively. In addition, we created a draft genome assembly based on the Illumina sequencing only for *G. caudata* (30 Mb) and reassembled the genome of *G. vermiculophylla* (Flanagan et al. 2021) to a final 45 Mb after bacterial contamination removal. The above genome assemblies were comparable with the genomes of *Gracilaria domingensis* (78 Mb; Nakamura-Gouvea et al. 2022) and *Gracilaria changii* (36 Mb; Ho et al. 2018). PacBio assemblies of *G. chilensis* and *G. gracilis* produced here (138 and 279 contigs, respectively; N50 of 1.56 and 0.56 Mb, respectively) are the most contiguous red macroalgal genomes presently available in public databases, apart from *G. vermiculophylla* and *Pyropia yezoensis* where the addition of a HiC library enabled scaffolding nearly at the chromosome level (Wang et al. 2020; Flanagan et al. 2021). In *G. vermiculophylla*, however, regardless of the high N50 of 2.56 Mb, the total number of contigs/scaffolds was also high (7,753/4,240). The *G. caudata* assembly was fragmented with a low N50 of 21 kb and 55,767/5,535 contigs/scaffolds. Despite the differences in assembly size, BUSCO scores were similar across the long-read–sequenced *G. gracilis* and *G. chilensis* (83.6% and 81.6% of conserved proteins present) and the more fragmented *G. caudata* genome (81.6%, Eukaryota_odb10; Manni et al. 2021, Simão et al. 2015; table 1). The reassembled genome of *G. vermiculophylla* contained 71.8% of the conserved proteins. Given the diversity of Rhodophyta and the lack of lineage-specific databases, these results are in the expected range. A recent study estimated the presence of conserved eukaryotic genes (Eukaryota_odb10) in red algal genomes at a median level of 69% (Hanschen et al. 2020).

**Table 1.** Assembly Statistics

	*G. chilensis*	*G. vermiculophylla*	*G. caudata*	*G. gracilis*
Strain	NLEC103-M9	HapMaleFtJ-2017	M-176_S67	GNS1m
Sequencing	PacBio	Illumina, HiC	Illumina	PacBio
Genome size	76.07 Mb	44.95 Mb	30.28 Mb	72.49 Mb
Contigs/scaffolds	138/138	7,753/4,240	55,767/5,535	279/279
GC contents	48.9%	49.5%	49.9%	46.6%
N50	1.56 Mb	2.56 Mb	20.8 kb	563 kb
L50	18	6	396	38
Repeat content	66.2%	48.3%	45.7%	60.7%
Protein-coding genes	7,943	6,807	8,737	9,460
Av. gene length	1,404 bp	1,751 bp	1,409 bp	1,643 bp
Genes w. interpro/Uniprot 90^a^	93.4%/88.8%	93.6%/89.7%	91.7%/86.5%	92.0%/84.2%
Genes with GO annotation	52.7%	54.4%	49.9%	47.9%
Genes with intron	23.4%	24.1%	28.6%	29.4%
BUSCO complete	75.3%	65.1%	73.0%	77.3%
BUSCO fragmented	6.3%	6.7%	8.6%	6.3%
BUSCO missing	18.4%	28.2%	18.4%	16.4%

a *e*-value cutoff 1e-5.

**Fig. 1.**
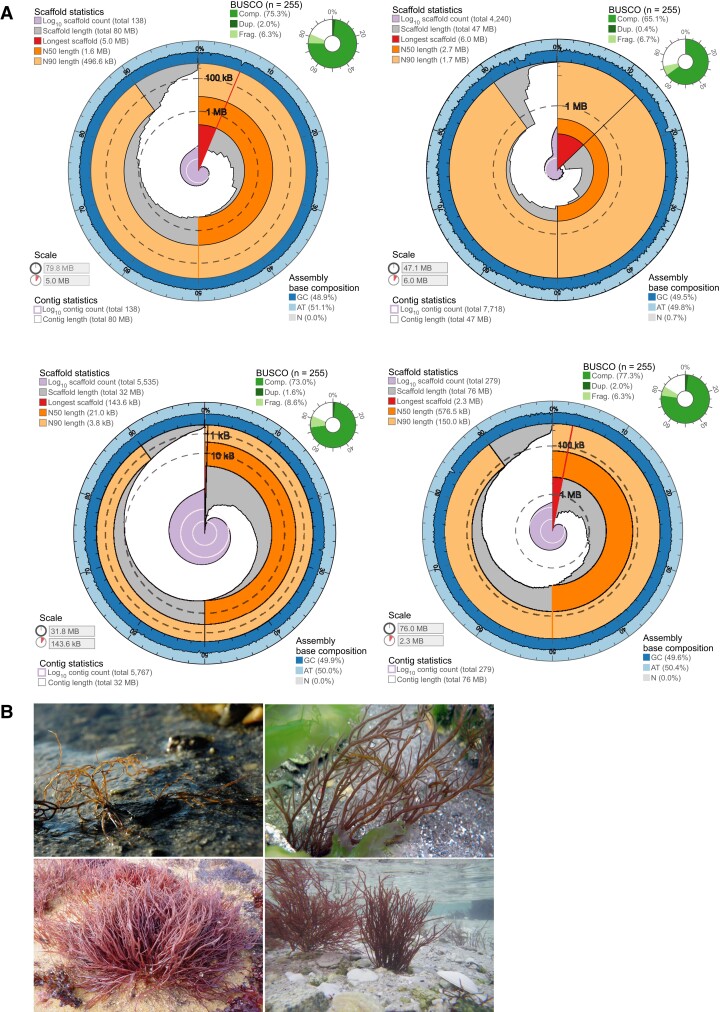
(*A*) Genome assembly metrics of *G. chilensis* (top left), *G. vermiculophylla* (top right), *G. caudata* (bottom left), and *G. gracilis* (bottom right) (Challis 2017; https://github.com/rjchallis/assembly-stats). The inner radius of the circular plot represents the length of the longest scaffold in the assembly and the proportion of the assembly that it represents. The cumulative number of scaffolds within a given percentage of the genome is plotted in light purple originating at the center of the plot. The N50 and N90 scaffold lengths are indicated by dark and light orange, respectively. Genome scaffolds are plotted in gray from the circumference and the length of segment at a given percentage indicates the cumulative percentage of the assembly that is contained within scaffolds of at least that length. The guanine–cytosine (GC) content is marked by the dark blue outer circle. Complete, fragmented, and duplicated BUSCO genes are shown in green in the upper right corner. (*B*) *Gracilaria chilensis* (top left), *G. vermiculophylla* (top right), *G. caudata* (bottom left), and *G. gracilis* (bottom right). Photo credit in order: M.-L. Guillemin, S. Krueger-Hadfield, E. M. Plastino, C. Destombe.

Red algal genomes are repeat rich, with half or more of their genomic sequence being constituted by repetitive elements, as reported previously for *Porphyra umbilicalis* (43.9%; Brawley et al. 2017), *P. yezoensis* (48%; Wang et al. 2020), and *Chondrus crispus* (73%; Collén et al. 2013). In agreement with this general trend, between 45.7% and 66.2% of the *Gracilaria* genomes corresponded to repetitive elements (fig. 1, supplementary fig. S2, Supplementary Material online, and table 1).

### Gene Prediction and Annotation

Gene prediction yielded a total of 7,943, 8,737, and 9,460 protein-coding sequences for *G. chilensis*, *G. caudata*, and *G. gracilis* (table 1), which was comparable with other red macroalgal genomes, *C. crispus* (9,815 genes; Collén et al. 2013) and *G. changii* genome (10,912 genes; Ho et al. 2018). In addition, we annotated the reassembled genome of *G. vermiculophylla*, which yielded fewer genes (6,807). Among these genes, 70.6–76.6% did not contain any introns, as typical for the compact genomes of red algae (Qiu et al. 2015). Most *Gracilaria* genes had homologous sequences in the Uniprot database (84.2–89.7%) and were annotated with at least one INTERPRO hit (91.7–93.6%). Between 47.9% and 54.4% of genes were associated with gene ontology (GO) annotations.

OrthoFinder analyses identified 4,666 orthogroups present in all four genomes (supplementary fig. S2, Supplementary Material online) versus 408–620 orthogroups or orphan genes specific to only one of the sequenced species (supplementary fig. S2, Supplementary Material online). Among the species-specific sequences, the rate of GO annotation was lower than for the entire data set, ranging from 12.7% for *G. chilensis* to 18.2% for *G. caudata*. Both the annotated and the unknown species-specific genes constitute attractive targets to study their role in adaptation and speciation.

### Rhodoexplorer Red Algal Genome Database

In addition to depositing the raw reads and sequenced genome in a public repository, we integrated the data into the newly created Rhodoexplorer Red Algal Genome Database (https://rhodoexplorer.sb-roscoff.fr), which will include more red algal genomes in the future. The services provided include the following:

- Information about the sequenced strains, with links to external databases (NCBI, WoRMS, and Algaebase).
- Assembly and annotation metrics.
- Data downloads: genomic, genes and proteomic data sets, structural and functional annotations, orthology clusters, etc.
- A Blast interface with a selection of red algal genomes, predicted and de novo assembled transcriptomes and proteomes.
- Visualization tools: a genome browser to visualize the predicted genes and the RNA-sequence (RNAseq) data mapped on the genome and a web interface to visualize functional annotations and retrieve individual protein sequences.

## Materials and Methods

### Sampling of the Biological Material

Adult female and male *Gracilaria* thalli, all bearing reproductive structures, were collected from natural populations: *G. chilensis* in Lenca (Chile, −41.607, −72.692), *G. vermiculophylla* in Charleston, SC (USA, 32.752, −79.900), *G. caudata* in Paracuru, CE (Brazil, −3.399, −39.012), and *G. gracilis* in Cape Gris-Nez (France, 50.872, 1.584). *Gracilaria caudata* and *G. chilensis* were maintained as clonal, unialgal cultures under laboratory conditions prior to nucleic acid extractions (see *Culture conditions*). Field-collected *G. gracilis* and *G. vermiculophylla* thalli were transported to the laboratory, examined under a microscope, and cleaned of contaminants. If visible, cystocarps were excised prior to preservation of the thalli at −80°C. Supplementary table S2, Supplementary Material online provides details of the *Gracilaria* species used in this study.

### Culture Conditions

Cultures were initiated either from lab crosses or from tetraspores released by field-collected tetrasporophytes. *Gracilaria caudata* was grown in the modified von Stosch nutrient solution (Ursi and Plastino 2001) diluted to 25% in seawater (32 practical salinity unit [psu]), with weekly renewals. The algae were kept in culture chambers at 25 °C under fluorescent illumination of 70 *μ*mol m^−2^ s^−1^ 14-h photoperiod, following previously established optimal growth conditions (Yokoya and Oliveira 1992a, 1992b). *Gracilaria chilensis* was grown in Provasoli medium (McLachlan 1973), changed weekly during the first 2 months and twice a week thereafter. Cultures were kept at 13 °C under 40–60 *μ*mol m^−2^ s^−1^ of light with 12-h day length.

### Nucleic Acid Extraction, Library Preparation, and Sequencing

Genomic DNA (gDNA) was extracted using DNeasy PowerPlant Pro Kit for *G. caudata* or an in-house protocol based on Faugeron et al. (2001) for *G. chilensis* and *G. gracilis*. The concentration and purity of DNA were measured with NanoDrop and Qubit before sequencing on an Illumina HiSeq 2500 (125-bp PE reads for *G. chilensis* and *G. gracilis*; 100-bp PE reads for *G. caudata*) or PacBio Sequel II with sheared gDNA large insert library (*G. gracilis* and *G. chilensis*) (supplementary table S2, Supplementary Material online).

For genome annotation, total RNA was extracted from mature thalli of male and female gametophytes of *G. chilensis*, *G. caudata*, and *G. gracilis* using the RNeasy Mini Plant Kit (Qiagen) and Macherey Nagel NucleoSpin RNA Plant Kit for *G. vermiculophylla*, following the manufacturer's instructions. Paired-end 150-bp Illumina reads were generated with Illumina HiSeq 2500 (supplementary table S2, Supplementary Material online).

### Genome Assembly

De novo genome assemblies for *G. gracilis* and *G. chilensis* were generated based on 203-fold and 116-fold coverage of PacBio long reads, respectively. Bacterial sequences were removed from raw data (subreads) using BlobTools v1.1.1 (Laetsch and Blaxter 2017). Two independent assemblies were generated using CANU (Koren et al. 2017) and FLYE (Kolmogorov et al. 2019). Based on congruity (QUAST v.5.0.2; Mikheenko, et al. 2018) and BUSCO score (Simão et al. 2015), the best assembly was kept and polished using three iterations of RACON v.1.4.20. Finally, PacBio sequencing error was corrected using 150-bp paired-end Illumina reads with PILON v.1.23 software (Walker et al. 2014). The draft genome assembly of *G. caudata* was generated using 171-fold coverage of 150-bp paired-end Illumina reads only. First, a meta-genome was produced using metaSPAdes v3.12.0 (Nurk et al. 2017) and bacterial contigs were detected using BlobTools. Reads corresponding to eukaryotic contigs were then assembled using SPAdes v3.12.0 (Bankevich et al. 2012).

For *G. vermiculophylla*, we updated the existing chromosome-scale genome assembly (Flanagan et al. 2021) by reassembling the Illumina reads using SPAdes v3.12.0 (Bankevich et al. 2012) and scaffolding with HiC libraries, following the Dovetail Genomics proprietary pipeline (Elbers et al. 2019). This process ameliorated the genome continuity (N50 increased from 2.06 to 2.68 Mb) and completeness (BUSCO score increased from 57.6% to 65.9% of complete genes using the Eukaryota_odb10 data set).

Genome assemblies were validated with a final BlobTools v1.1.1 analysis (Laetsch and Blaxter 2017) using DNAseq mapping coverage files produced by HISAT2 v2.2.1 (Kim et al. 2019), Diamond BlastX v2.0.11 (Buchfink et al. 2015, 2021) hit-file against nonredundant protein sequences archive from NCBI (-sensitive, –max-target-seqs 1, -e-value 1e-20), and Blast v2.12.0 (Camacho et al. 2009) output against nucleotide archive from NCBI (-max_target_seqs 10 -max_hsps 1 -evalue 1e-20) as input genomic scaffolds classified as bacterial or with a coverage of <1 (sum of coverages for each sequence across all coverage files) were removed from the assembly. Genome assembly completeness was assessed using BUSCO scores with the eukaryotic data set (Eukaryota_odb10; Simão et al. 2015; Manni et al. 2021).

Chloroplastic and mitochondrial genomes of each species were reconstructed from Illumina raw reads using NOVOPlasty (Dierckxsens et al. 201 7) through the European Galaxy web portal (https://usegalaxy.eu/). Annotation of those de novo organellar genomes was done using the GeSeq web tool (Tillich et al. 2017; https://chlorobox.mpimp-golm.mpg.de/geseq.html). Public sequences from *G. caudata* voucher SPF:57390 (NC_039146, NC_039139), *G. chilensis* voucher CNU050183 (KP728466, KT266788), *G. gracilis* voucher SPF:55734 (NC_039141, NC_039148), and *G. vermiculophylla* (MN853882, MH396022) were retrieved from NCBI and used as seeds and references for both assembly and annotation.

### Genome Annotation

Each reference genome was first masked using RepeatMasker v4.0.9 (Smit et al. 2013–2015) with Dfam v3.0 database (Wheeler et al. 2013) and a customized repeat library produced from concatenated outputs of RepeatScout v1.0.6 (Price et al. 2005) and TransposonPSI v1.0.0 (Haas 2007–2011). Initial quality assessment of the RNAseq reads was performed with FastQC v0.11.9 (Andrews 2010), and reads were trimmed using Trimmomatic v0.39 (TRAILING:3 SLIDINGWINDOW:4:15 MINLEN:50; Bolger et al. 2014). Clean reads were mapped to the reference genome assembly using HISAT2 v2.2.1 (Kim et al. 2019) and used to annotate protein-coding genes with BRAKER2 v2.1.6 (Bruna et al. 2021). Functional annotation of the transcriptomes was performed using eggNOG-mapper (Huerta-Cepas et al. 2019; Cantalapiedra et al. 2021).

All codes used for genomes assembly and annotation are available on the Gitpage dedicated to the genome database project https://abims-sbr.gitlab.io/rhodoexplorer/doc/data_process/.

### Rhodoexplorer Red Algal Genome Database

The main web portal (https://rhodoexplorer.sb-roscoff.fr) has been implemented using the Python web framework Django, with data stored in a relational database (PostgreSQL).

For each red algal species, an integrated environment of visualization tools has been deployed based on the Galaxy Genome Annotation (GGA) project (Bretaudeau et al. 2019). Each GGA environment deployed for the Rhodoexplorer database includes the following: Chado, a PostgreSQL relational database schema for storing biological data (Mungall et al. 2007); JBrowse, a web-based genome browser (Buels et al. 2016); Tripal, a Drupal-based application for creating biological websites (Sanderson et al. 2013); Elasticsearch, a distributed, free, and open search and analytics engine for all types of data (https://www.elastic.co/products/elasticsearch); and Galaxy, a browser-accessible workbench for scientific computing used as a data loading orchestrator for administrators (The Galaxy Community 2022). To facilitate the deployment and the administration of the GGA service, a set of Python tools has been developed (http://gitlab.sb-roscoff.fr/abims/e-infra/gga_load_data) allowing mass deployment of Docker containers and automated data loading through Galaxy with the Bioblend API (Sloggett et al 2013).

The Blast interface (https://blast.sb-roscoff.fr/rhodoexplorer/) includes an implementation of the Blast algorithm using SequenceServer (Priyam et al. 2019) graphical.

The documentation website for navigating the platform web portal and resources (https://abims-sbr.gitlab.io/rhodoexplorer/doc/) is published from a GitLab repository, with Pages and MkDocs, a static site generator.

The entire informatic infrastructure is deployed and maintained on the ABiMS Bioinformatics platform of the Roscoff Biological Station, part of the national infrastructure French Bioinformatic Institute.

## Supplementary Material

evad124_Supplementary_Data

## Data Availability

Sequencing data have been deposited in the SRA database under BioProjects PRJNA936482, PRJNA931233, PRJNA938301, and PRJNA938403. The accession numbers for the raw sequence data are provided in supplementary table S2, Supplementary Material online. *Gracilaria chilensis*, *G. gracilis*, and *G. caudata* Whole Genome Shotgun project have been deposited at DDBJ/ENA/GenBank under the accessions JARGXX000000000, JARGSG000000000, and JASCIV000000000, respectively. *Gracilaria vermiculophylla* updated assembly has been deposited under JAHNZQ000000000.
