# Supplementary material for: The Rhodoexplorer Platform for Red Algal Genomics and Whole-Genome Assemblies for Several Gracilaria Species
Source: Genome Biol Evol. 2023 Jul 22;15(7):evad124. doi: 10.1093/gbe/evad124 (PMC10388701; doi:10.1093/gbe/evad124)
Supplement: evad124_Supplementary_Data [file evad124_supplementary_data.zip › GBE_bioRxiv_waccno_SUPPFIGS_clean.docx]

THE RHODOEXPLORER PLATFORM FOR RED ALGAL GENOMICS AND WHOLE GENOME ASSEMBLIES FOR SEVERAL GRACILARIA SPECIES

Agnieszka P. Lipinska^1,3#,*^, Stacy A. Krueger-Hadfield^2,#,*,!^, Olivier Godfroy^3^, Simon Dittami^3^, Lígia Ayres-Ostrock^4,5^, Guido Bonthond^6^, Loraine Brillet-Guéguen^3,7^, Susana Coelho^1^, Erwan Corre^7^, Guillaume Cossard^1^, Christophe Destombe^8^, Paul Epperlein^1^, Sylvain Faugeron^8,9^, Elizabeth Ficko-Blean^3^, Jessica Beltrán^8,9^, Emma Lavaut^8^, Arthur Le Bars^7,10^, Fabiana Marchi^4^, Stéphane Mauger^8^, Gurvan Michel^3^, Philippe Potin^3^ , Delphine Scornet^3^, Erik E. Sotka^11^, Florian Weinberger^12^, Mariana Cabral de Oliveira^4^, Marie-Laure Guillemin^8,13,14^, Estela M. Plastino^4^, Myriam Valero^8^

^1^ Department of Algal Development and Evolution, Max Planck Institute for Biology Tubingen, Tubingen, Germany

^2^ Department of Biology, University of Alabama at Birmingham, 1300 University Blvd, Birmingham, AL, 35294

^3^ Sorbonne Université, CNRS, UMR 8227, Laboratory of Integrative Biology of Marine Models, Station Biologique de Roscoff, Roscoff, France

^4^ Departamento de Botânica, Instituto de Biociências, Universidade de São Paulo, Rua do Matão 277, Cidade Universitária 05508-090, São Paulo, SP, Brasil.

^5^ Hortimare - Breeding & Propagating Seaweed. Altonstraat 25A 1704 CC Heerhugowaard. The Netherlands

^6^ Institute for Chemistry and Biology of the Marine Environment (ICBM), Carl von Ossietzky University Oldenburg, Schleusenstrasse 1, 26382, Wilhelmshaven, Germany

^7^ CNRS, Sorbonne Université, FR2424, ABiMS-IFB, Station Biologique, 29680, Roscoff, France

^8^ CNRS, Sorbonne Université, Pontificia Universidad Católica de Chile, Universidad Austral de Chile, IRL 3614, Evolutionary Biology and Ecology of Algae, Station Biologique de Roscoff, CS 90074, F-29688 Roscoff, France

^9^ Núcleo Milenio MASH, Facultad de Ciencias Biológicas, Pontificia Universidad Católica de Chile, Santiago, Chile

^10^ CNRS, Institut Français de Bioinformatique, IFB-core, UMS 3601, Évry, France

^11^ Department of Biology, College of Charleston, Charleston SC 29412

^12^ GEOMAR Helmholtz-Zentrum für Ozeanforschung, Marine Ecology Division, Düsternbrooker Weg 20, 24105 Kiel, Germany

^13^ Núcleo Milenio MASH, Facultad de Ciencias, Instituto de Ciencias Ambientales y Evolutivas, Universidad Austral de Chile, Casilla 567, Valdivia, Chile

^14^ Centro FONDAP de Investigación de Ecosistemas Marinos de Altas Latitudes (IDEAL), Valdivia, Chile

^#^ Shared first authors

! Current address: Virginia Institute of Marine Science, Eastern Shore Laboratory, 40 Atlantic Ave, Wachapreague, VA 23480, [sakh@vims.edu](mailto:sakh@vims.edu)

* Authors for correspondence: **Agnieszka P. Lipinska**, Department of Algal Development and Evolution, Max Planck Institute for Developmental Biology, Tuebingen, Germany, +49-7071-601-1370, [alipinska@tuebingen.mpg.de](mailto:alipinska@tuebingen.mpg.de); **Stacy A. Krueger-Hadfield**, Department of Biology, University of Alabama at Birmingham, 1300 University Blvd, Birmingham, AL, 35294, +1-205-934-6034, [sakh@uab.edu](mailto:sakh@uab.edu) & Virginia Institute of Marine Science, Eastern Shore Laboratory, 40 Atlantic Ave, Wachapreague, VA 23480, [sakh@vims.edu](mailto:sakh@vims.edu)

*SUPPLEMENTARY MATERIAL*

Supplementary Figure S1: Life cycle of *Gracilaria.*

Supplementary Figure S2: Venn diagram of shared and species-specific orthogroups.

Fig. S1. Life cycle of *Gracilaria.* The life cycle consists of an alternation between haploid dioecious gametophytes and a diploid tetrasporophyte. The tetrasporophyte produces meiospores through meiosis, which develop as gametophytes after release. The sex of the gametophytes is determined by haploid sex chromosomes (UV system). Spores that receive the V sex chromosome develop as male gametophytes whereas spores that carry U chromosome will produce female gametophytes. After fertilization, the zygote develops within the carposporophyte on the female gametophyte and is mitotically amplified—producing thousands of diploid carpospores that after release will give rise to tetrasporophytes.

Fig. S2. Venn diagram of shared and species-specific orthogroups and orphan genes among the four sequenced Gracilaria species.

**Supplementary Figure S1:** Life cycle of *Gracilaria*.
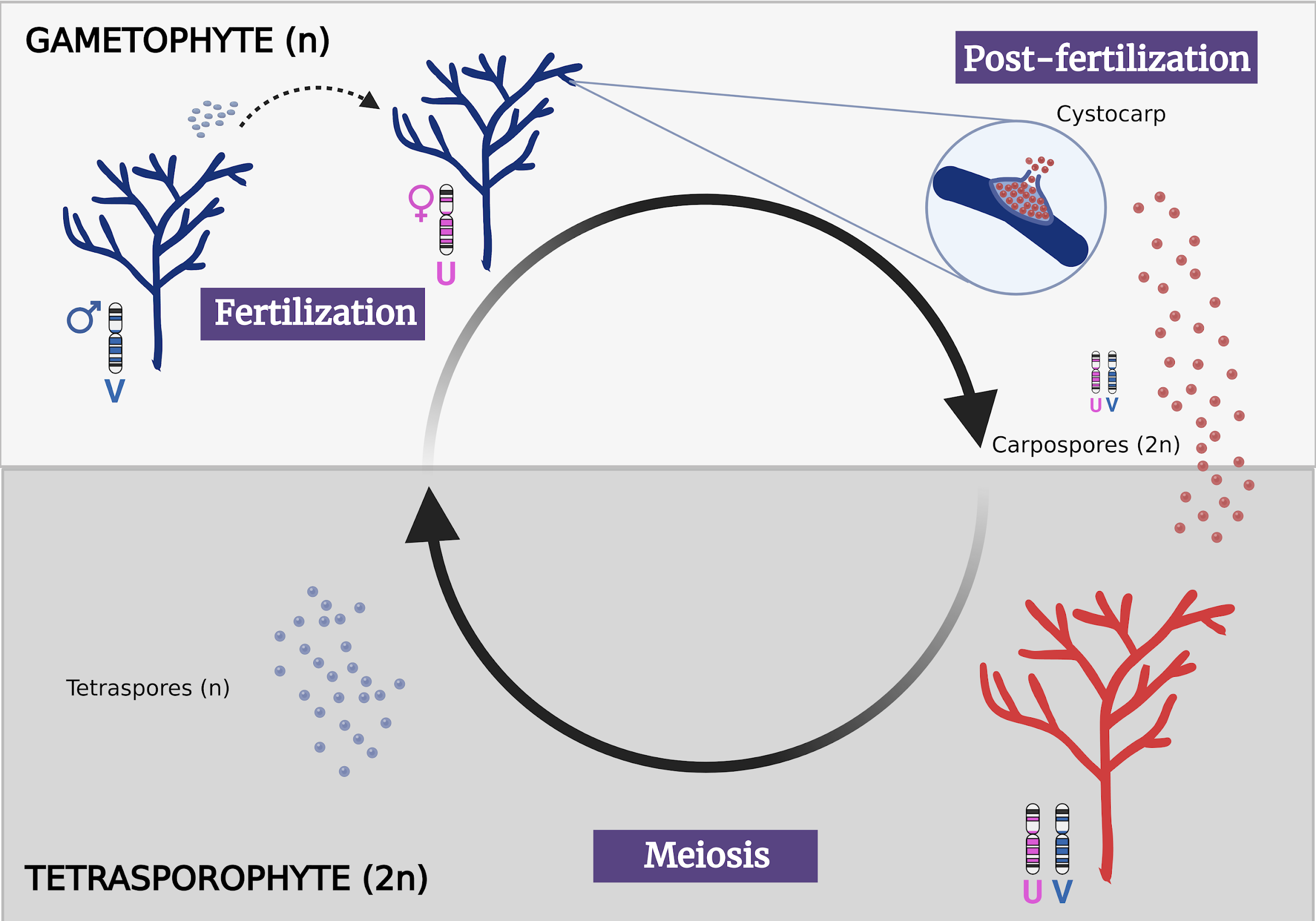


**
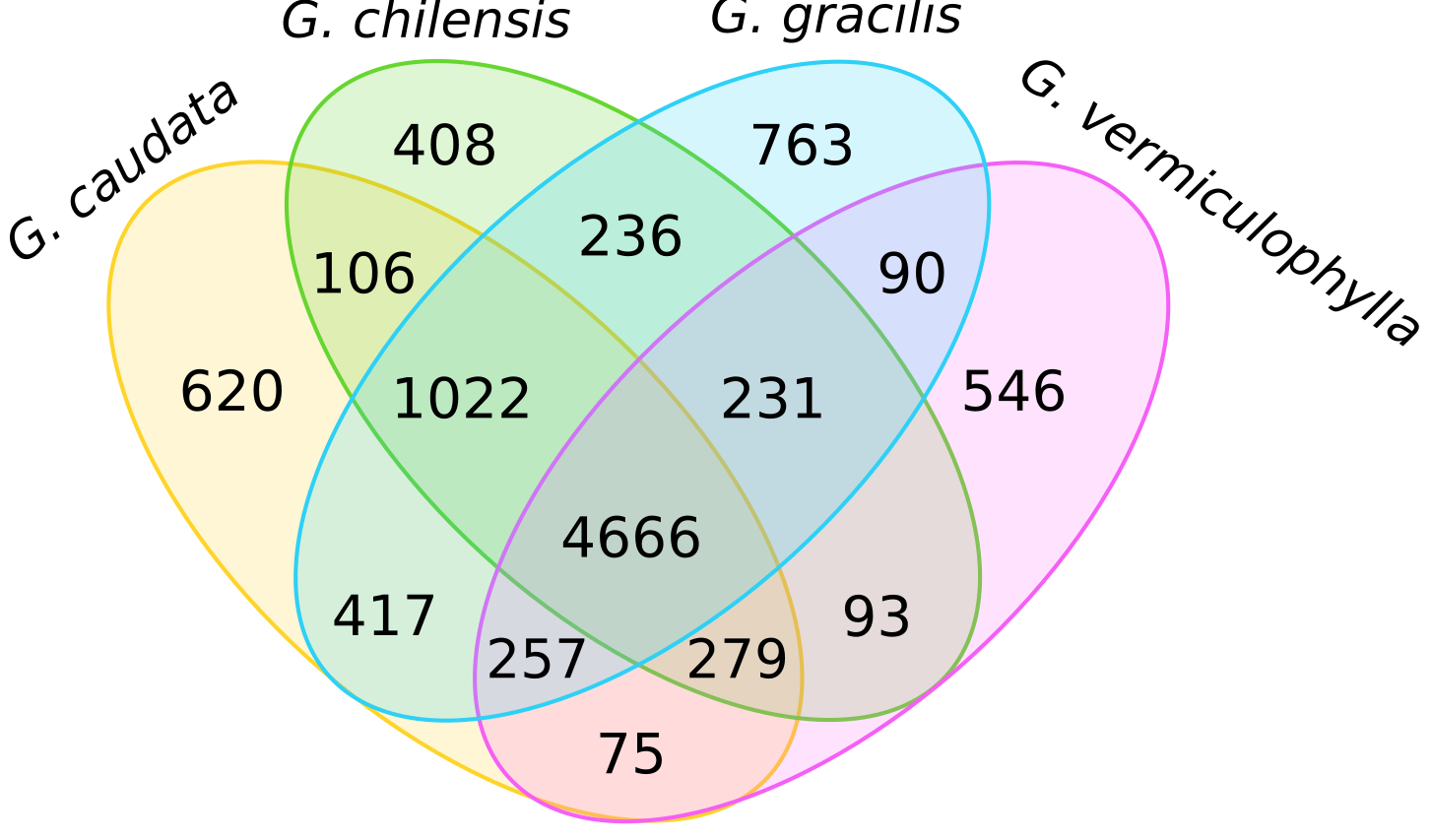
**

**Supplementary Figure S2:** Venn diagram of shared and species-specific orthogroups.
