# Supplementary material for: The Rhodoexplorer Platform for Red Algal Genomics and Whole-Genome Assemblies for Several Gracilaria Species
Source: Genome Biol Evol. 2023 Jul 22;15(7):evad124. doi: 10.1093/gbe/evad124 (PMC10388701; doi:10.1093/gbe/evad124)
Supplement: evad124_Supplementary_Data [file evad124_supplementary_data.zip › GBE_bioRxiv_waccno_SUPPTABLES.docx]

THE RHODOEXPLORER PLATFORM FOR RED ALGAL GENOMICS AND WHOLE GENOME ASSEMBLIES FOR SEVERAL GRACILARIA SPECIES

Agnieszka P. Lipinska^1,3#,*^, Stacy A. Krueger-Hadfield^2,#,*,!^, Olivier Godfroy^3^, Simon Dittami^3^, Lígia Ayres-Ostrock^4,5^, Guido Bonthond^6^, Loraine Brillet-Guéguen^3,7^, Susana Coelho^1^, Erwan Corre^7^, Guillaume Cossard^1^, Christophe Destombe^8^, Paul Epperlein^1^, Sylvain Faugeron^8,9^, Elizabeth Ficko-Blean^3^, Jessica Beltrán^8,9^, Emma Lavaut^8^, Arthur Le Bars^7,10^, Fabiana Marchi^4^, Stéphane Mauger^8^, Gurvan Michel^3^, Philippe Potin^3^ , Delphine Scornet^3^, Erik E. Sotka^11^, Florian Weinberger^12^, Mariana Cabral de Oliveira^4^, Marie-Laure Guillemin^8,13,14^, Estela M. Plastino^4^, Myriam Valero^8^

^1^ Department of Algal Development and Evolution, Max Planck Institute for Biology Tubingen, Tubingen, Germany

^2^ Department of Biology, University of Alabama at Birmingham, 1300 University Blvd, Birmingham, AL, 35294

^3^ Sorbonne Université, CNRS, UMR 8227, Laboratory of Integrative Biology of Marine Models, Station Biologique de Roscoff, Roscoff, France

^4^ Departamento de Botânica, Instituto de Biociências, Universidade de São Paulo, Rua do Matão 277, Cidade Universitária 05508-090, São Paulo, SP, Brasil.

^5^ Hortimare - Breeding & Propagating Seaweed. Altonstraat 25A 1704 CC Heerhugowaard. The Netherlands

^6^ Institute for Chemistry and Biology of the Marine Environment (ICBM), Carl von Ossietzky University Oldenburg, Schleusenstrasse 1, 26382, Wilhelmshaven, Germany

^7^ CNRS, Sorbonne Université, FR2424, ABiMS-IFB, Station Biologique, 29680, Roscoff, France

^8^ CNRS, Sorbonne Université, Pontificia Universidad Católica de Chile, Universidad Austral de Chile, IRL 3614, Evolutionary Biology and Ecology of Algae, Station Biologique de Roscoff, CS 90074, F-29688 Roscoff, France

^9^ Núcleo Milenio MASH, Facultad de Ciencias Biológicas, Pontificia Universidad Católica de Chile, Santiago, Chile

^10^ CNRS, Institut Français de Bioinformatique, IFB-core, UMS 3601, Évry, France

^11^ Department of Biology, College of Charleston, Charleston SC 29412

^12^ GEOMAR Helmholtz-Zentrum für Ozeanforschung, Marine Ecology Division, Düsternbrooker Weg 20, 24105 Kiel, Germany

^13^ Núcleo Milenio MASH, Facultad de Ciencias, Instituto de Ciencias Ambientales y Evolutivas, Universidad Austral de Chile, Casilla 567, Valdivia, Chile

^14^ Centro FONDAP de Investigación de Ecosistemas Marinos de Altas Latitudes (IDEAL), Valdivia, Chile

^#^ Shared first authors

! Current address: Virginia Institute of Marine Science, Eastern Shore Laboratory, 40 Atlantic Ave, Wachapreague, VA 23480, [sakh@vims.edu](mailto:sakh@vims.edu)

* Authors for correspondence: **Agnieszka P. Lipinska**, Department of Algal Development and Evolution, Max Planck Institute for Developmental Biology, Tuebingen, Germany, +49-7071-601-1370, [alipinska@tuebingen.mpg.de](mailto:alipinska@tuebingen.mpg.de); **Stacy A. Krueger-Hadfield**, Department of Biology, University of Alabama at Birmingham, 1300 University Blvd, Birmingham, AL, 35294, +1-205-934-6034, [sakh@uab.edu](mailto:sakh@uab.edu) & Virginia Institute of Marine Science, Eastern Shore Laboratory, 40 Atlantic Ave, Wachapreague, VA 23480, [sakh@vims.edu](mailto:sakh@vims.edu)

*SUPPLEMENTARY MATERIAL*

Supplementary Table S1: Available red algal genomic resources.

Supplementary Table S2: Species used in this study.

**Supplementary Table S1:** Available red algal wholes genome sequences. M=multicellular, U=unicellular.

| **Species** | **Order** | **N50** | **U/M** | **Citation** |
| --- | --- | --- | --- | --- |
| *Chondrus crispus* | Gigartinales | 250kb | M | https://doi.org/10.1073/pnas.1221259110 |
| *Galdieria sulphuraria* | Cyanidiales | 230kb | U | https://doi.org/10.7554/eLife.45017 |
| *Galdieria phlegrea* | Cyanidiales | 201kb | U | https://doi.org/10.7554/eLife.45017 |
| *Gracilaria changii* | Gracilariales | 17kb | M | https://doi.org/10.1016/j.ygeno.2017.09.003 |
| *Gracilaria domingensis* | Gracilariales | 189kb | M | https://doi.org/10.1111/jpy.13238 |
| *Gracilaria vermiculophylla* | Gracilariales | 2Mb | M | https://doi.org/10.1111/mec.15854 |
| *Gracilariopsis chorda* | Gracilariales | 220kb | M | https://doi.org/10.1093/molbev/msy081 |
| *Gracilariopsis lemaneiformis* | Gracilariales | 35kb | M | https://doi.org/10.1186/s12870-018-1309-2 |
| *Calliarthron tuberculosum* | Corallinales | n/a | M | https://doi.org/10.1016/j.cub.2011.01.037 |
| *Porphyridium purpureum* | Porphyridiales | 20kb | U | https://doi.org/10.1038/ncomms2931 |
| *Porphyra umbilicalis* | Bangiales | 202kb | M | https://doi.org/10.1073/pnas.1703088114 |
| *Neoporphyra haitanensis* | Bangiales | 650kb | M | https://doi.org/10.1093/molbev/msab315 |
| *Neopyropia yezoensis* | Bangiales | 34Mb | M | https://doi.org/10.1038/s41467-020-17689-1 |
| *Kappaphycus alvarezii* | Gigartinales | 849kb | M | https://doi.org/10.1101/2020.02.15.950402 |
| *Asparagopsis taxiformis* | Bonnemaisoniales | 2kb | M | https://doi.org/10.1021/acschembio.0c00299 |
| *Cyanidium caldarium* | Cyanidiales | 13kb | U | https://www.ncbi.nlm.nih.gov/genome/7354* |
| *Cyanidiococcus yangmingshanensis* | Cyanidiales | 653kb | U | https://doi.org/10.1111/jpy.13056 |
| *Cyanidioschyzon merolae* | Cyanidioschyzonales | 846kb | U | https://doi.org/[10.1186/1741-7007-5-28](https://doi.org/10.1186/1741-7007-5-28)  https://doi.org/[10.1038/nature02398](https://doi.org/10.1038/nature02398) |

* no publication associated
n/a data no longer accessible

**Supplementary Table S2:** Species used in this study

| Species | Strain name | Isolation location | Sex | Type of data | **Accession numbers** |
| --- | --- | --- | --- | --- | --- |
| *Gracilaria chilensis* | NLEC103-F17 | Lenca, Region of Puerto Montt, Chile (-41.607, -72.692) | Female | RNAseq | SRR23519128 |
|  | NLEC103-F17 |  | Female | DNAseq | SRR23519124 |
|  | NLEC103-F20 |  | Female | RNAseq | SRR23519127 |
|  | NLEC103-F20 |  | Female | DNAseq | SRR23519123 |
|  | NLEC103-M9 |  | Male | RNAseq | SRR23519129 |
|  | NLEC103-M9 |  | Male | DNAseq | SRR23519122, SRR23519125 |
|  | NLEC103-M1 |  | Male | RNAseq | SRR23519130 |
|  | NLEC103-M2 |  | Male | DNAseq | SRR23519126 |
| *Gracilaria gracilis* | GNS1m | Cap-Gris-Nez Northen France (50.872, 1.584) | Male | DNAseq | SRR23565662, SRR23565669 |
|  | GNS1m |  | Male | RNAseq | SRR23565661 , SRR23565660, SRR23565659, SRR23565670 |
|  | GNS1f |  | Female | DNAseq | SRR23565672, SRR23565663 |
|  | GNS1f |  | Female | RNAseq | SRR23565671, SRR23565666 , SRR23565665 , SRR23565664 |
|  | GNH218m |  | Male | DNAseq | SRR23565667 |
|  | GNH47aAf |  | Female | DNAseq | SRR23565668 |
| *Gracilaria caudata* | 172F | Pedra Rachada beach, Paracuru, Ceará, Brazil (-3.399, -39.012) | Female | DNAseq | SRR23610505 |
|  | 172F |  | Female | RNAseq | SRR23610508 |
|  | 174F |  | Female | DNAseq | SRR23610506 |
|  | 174F |  | Female | RNAseq | SRR23610509 |
|  | 176M |  | Male | DNAseq | SRR23610514 |
|  | 176M |  | Male | RNAseq | SRR23610511 |
|  | 178M |  | Male | DNAseq | SRR23610513 |
|  | 178M |  | Male | RNAseq | SRR23610515 |
|  | 179M |  | Male | RNAseq | SRR23610516 |
|  | 177M |  | Male | RNAseq | SRR23610512 |
|  | 175F |  | Female | RNAseq | SRR23610510 |
|  | 171F |  | Female | RNAseq | SRR23610507 |
| *Gracilaria vermiculophylla* | Gver_F | Charleston, SC, USA (32.752, -79.900) | Female | DNAseq | SRR23609120 |
|  | fjs03mal |  | Male | RNAseq | SRR23609119 |
|  | fjs33mal |  | Male | RNAseq | SRR23609118 |
|  | fjs36mal-New |  | Male | RNAseq | SRR23609117 |
|  | Fjs50mal-New |  | Male | RNAseq | SRR23609116 |
|  | fjs34fem |  | Female | RNAseq | SRR23609115 |
|  | fjs39fem |  | Female | RNAseq | SRR23609114 |
|  | fjs40fem |  | Female | RNAseq | SRR23609113 |
|  | fjsfem |  | Female | RNAseq | SRR23609112 |
