# Supplementary figures and images for: The Rhodoexplorer Platform for Red Algal Genomics and Whole-Genome Assemblies for Several Gracilaria Species
Source: Genome Biol Evol. 2023 Jul 22;15(7):evad124. doi: 10.1093/gbe/evad124 (PMC10388701; doi:10.1093/gbe/evad124)

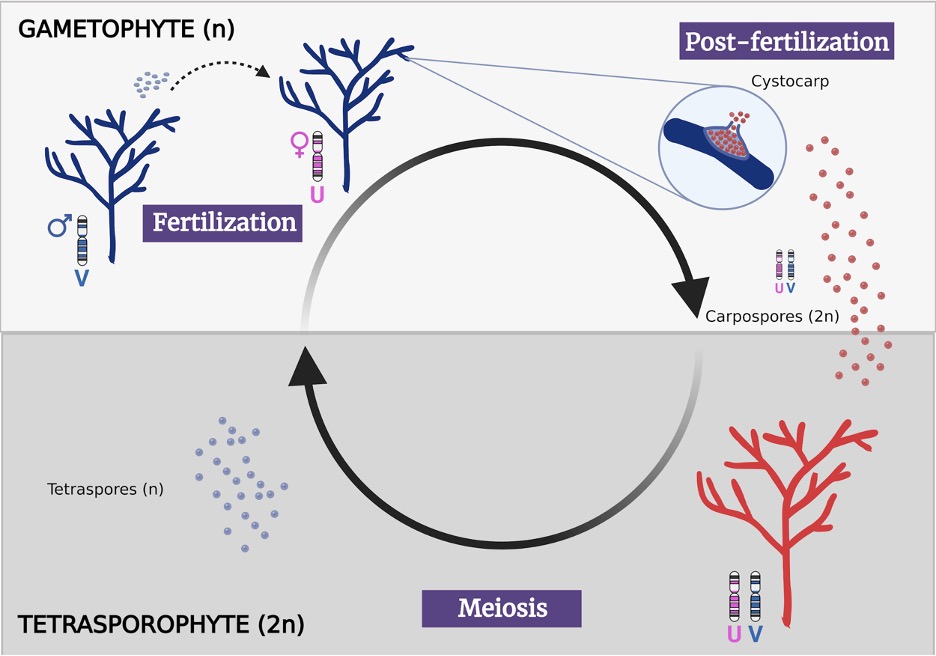

Supplement: evad124_Supplementary_Data [file evad124_supplementary_data.zip › FigureS1.jpg]
